# A preliminary report on applying the Schroth method principle after surgical fusion for scoliosis in a 23-year-old female with adolescent idiopathic scoliosis: a case report

**DOI:** 10.1186/1748-7161-8-S2-O9

**Published:** 2013-09-18

**Authors:** Berdishevsky Hagit

**Affiliations:** 1 Hospital for Special Surgery, New York, NY, USA

## Background

The Schroth method has proven to be an effective treatment for patients suffering from adolescent idiopathic scoliosis (AIS); however, its impact on patients who have undergone surgical intervention to correct scoliosis and who suffer from postural imbalances along with diminished respiration is unknown.

## Purpose

The aim of this study was to examine the effects of Schroth therapy on a 21-year-old female with AIS after spinal fusion.

## Methods

During an evaluation in December 2011, one month after spinal fusion (T10-Pelvic), the patient's clinical sagittal profile demonstrated a significant forward trunk inclination of 5 cm. Angle of Trunk Rotation (ATR) was 5° in the thoracic and 4° in the lumbar. Quality-of-life score was 3.3 (SRS 22 questionnaire). Body image was 3 (Trunk Appearance Perception Scale (TAPS)). Pain score was 4 using the Visual Analog Scale (VAS). Force vital capacity (FVC) was 2.1 liters. Chest expansion was 1.9 cm in the subaxillary, 2.2 cm in the nipple line and -2.0 cm around the waist. Positive bilateral Thomas Test for hip flexors and knee extensors. Gluteus maximus and medius strength -4/5 bilateral, quadratus lumbarum -3/5 bilaterally. Decreased abdominal and trunk extensor strength. Single-leg stance was 5 seconds right and 6 seconds left.

The physical therapy regimen included modified application of the Schroth exercises for patient with spinal fusion. The patient trained three 1-hour sessions per week for a duration of 10 weeks at an outpatient clinic, and was instructed to apply the learned principles during her daily activities and home exercise program, which consisted of 30-minute sessions five days per week. The patient is currently in treatment.

## Results

After the 10 week treatment period, the patient had demonstrated preliminary measurable improvement. The clinical trunk inclination reduced to 2.5 cm, the ATR had decreased to 3° in the thoracic and 2° in the lumbar. Quality-of-life score and body image showed improvement with a score of 4.5 on the SRS 22 and 5 on the TAPS. Pain score diminished to 1. FVC increased to 2.8 liters and chest expansion to 2.8 cm, 3.1 cm and 1 cm at measured locations. Gluteus maximus and medius bilaterally +4/5 and hip flexors and knee extensors improved in flexibility. The patient felt more comfortable with her appearance and reported satisfaction with the results.

## Conclusions and discussion

These preliminary findings may suggest that physical therapy utilizing the Schroth Method, with modification for fusion, may be a useful way to treat patients after spinal fusion.
